# Assessing Textbook Oncologic Outcomes in Distal Pancreatectomy for Pancreatic Adenocarcinoma: A National Cancer Database Study

**DOI:** 10.3390/jcm15103967

**Published:** 2026-05-21

**Authors:** Ahmed Alnajar, Jack Dalton Sleeman, Elif Zeynep Nerez, Mehmet Akcin, Danny Sleeman, Onur Kutlu

**Affiliations:** DeWitt Daughtry Department of Surgery, University of Miami Health System, Miami, FL 33136, USAokutlu@med.miami.edu (O.K.)

**Keywords:** pancreatic adenocarcinoma, distal pancreatectomy, textbook oncologic outcomes, pancreatic surgery, National Cancer Database, tail of pancreas, quality metrics

## Abstract

**Background**: This study investigates textbook oncologic outcomes (TOO), a measurement operationally defined to produce a holistic measure of surgical success, with respect to patients diagnosed with pancreatic adenocarcinoma undergoing distal (left) pancreatectomy for pancreatic adenocarcinoma. This study aims to identify factors associated with achieving TOO, emphasizing the role of hospital type. **Methods**: The NCDB (2010–2022) was queried for patients with clinical stage I–III pancreatic adenocarcinoma. Inclusion criteria consisted of patients > 18 who underwent curative partial or total pancreatectomy. The primary outcome was the achievement of TOO—operationally defined as R0 resection, ≥12 lymph nodes examined, no prolonged hospital stay, absence of 30-day mortality, and no readmissions. Logistic regression analyses were conducted to identify predictors of TOO. **Results**: Analysis of 11,194 patients showed that 38.9% achieved TOO. Achievement of TOO was associated with a median increase in one year in overall survival. Factors associated with TOO achievements in the adjusted model include female sex, private insurance, a lower Charlson/Deyo score, minimally invasive surgery (MIS), and high-volume centers. Notably, MIS emerged as a significant factor associated with 26% higher TOO (OR 1.26, 95% CI: 1.14–1.40) while treatment at high-volume hospitals was associated with 28–112% increased TOO (OR 1.28, 95% CI: 1.08–1.54 for Q3 volume and OR 2.12, 95% CI: 1.76–2.55 for Q4 volume). **Conclusions**: Achieving TOO is significantly influenced by patient demographics, clinical characteristics, and notably, the case volume of the treatment facility. These findings underscore the importance of considering centers experienced in surgical planning and patient counseling to optimize outcomes in distal pancreatectomies.

## 1. Introduction

Pancreatic adenocarcinoma represents one of the most challenging malignancies to treat, characterized by its insidious onset, aggressive nature, and poor prognosis. With a five-year survival rate that remains dismally low [1], the disease poses significant challenges for both clinicians and researchers alike. Early diagnosis is critical, as detection at a stage where the malignancy can be feasibly resected is the most important determinant of long-term survival; unfortunately, most patients present with advanced disease, limiting curative options [2]. Surgical resection offers the only potential for cure in the early stages of pancreatic cancer, underscoring the importance of optimizing surgical outcomes to improve patient survival and quality of life. Left-sided pancreatectomies, the surgical removal of the body and tail of the pancreas, are the cornerstone treatment for tumors located in these regions. In this work, the classification of a left-sided pancreatectomy is equivalent to that of a distal pancreatectomy [3]. The complexity of pancreatic surgeries and the high risk of postoperative complications necessitate a comprehensive evaluation of surgical success beyond traditional measures such as morbidity and mortality rates.

The concept of textbook oncologic outcomes (TOO) has emerged as a composite measure that provides a more holistic assessment of surgical success, incorporating factors such as margin-negative resection, adequate lymph node examination, absence of prolonged hospital stay, no 30-day mortality, and no readmissions [4,5]. Beyond operative performance, emerging evidence suggests that broader factors, including social determinants of health, may influence the likelihood of survival [5]. However, TOO reflects not only the technical success of the surgery but also the effective delivery of perioperative and oncologic care, offering a valuable benchmark for quality in pancreatic cancer surgery. Recent studies have begun to explore the factors influencing TOO in pancreatic surgeries, revealing insights into how patient demographics, tumor characteristics, and surgical techniques impact the achievement of these optimal outcomes.

The role of hospital type and the utilization of minimally invasive surgery (MIS) techniques have gained attention for their potential to improve TOO. MIS, including laparoscopic and robotic-assisted techniques, has emerged as the gold standard for distal pancreatectomies [6], largely replacing open surgery due to its advantages associated with reduced postoperative pain, shorter hospital stays, and quicker recovery times. These benefits suggest a promising avenue for enhancing surgical outcomes in pancreatic cancer treatment. However, the literature remains divided, with studies yielding mixed results on the benefits of MIS in achieving TOO in distal pancreatectomy. This discrepancy underscores the need for further research to identify the relationship between the choice of surgical modality and oncologic outcomes in this patient population.

Given the significant implications of achieving TOO on patient survival and quality of life, identifying factors that contribute to optimal surgical outcomes is critical. This study aims to fill a gap in the existing literature by examining the role of hospital type and the use of minimally invasive approaches in achieving TOO in patients undergoing distal pancreatectomy for pancreatic adenocarcinoma.

## 2. Methods

### 2.1. Data Source and Study Population

This study’s data were sourced from the comprehensive and de-identified 2022 National Cancer Database (NCDB) Participant User Data File, which compiles patient information across more than 1500 hospitals accredited by the Commission on Cancer in the United States. This robust database captures around 70% of all newly diagnosed cancer cases nationwide annually, making it a valuable resource for epidemiological and outcomes research in oncology.

**Figure 1:**
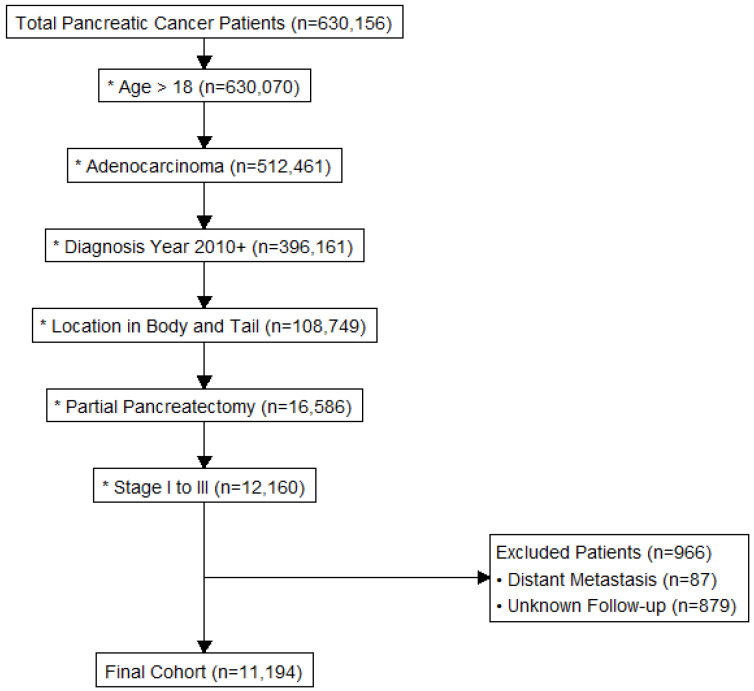
CONSORT diagram for the inclusion and exclusion criteria. * Indicate an inclusion criterion.

For the purposes of our study, we specifically included adult patients > 18 years with a diagnosis of pancreatic adenocarcinoma. Eligible patients had tumors located in the body or tail of the pancreas and underwent curative intent partial pancreatectomy. Only cases diagnosed after 1 January 2010 were considered, ensuring contemporary treatment modalities and surgical techniques were captured. Patients who did not meet staging inclusion criteria were excluded, including those with Stage 0/in situ disease (N = 3832), Stage IV disease (N = 106,913), or unknown pathological stage (N = 407,122). Other patients were excluded from the study if they were known to have distant metastasis on clinical or pathological staging (N = 87) or had unknown follow-up data (N = 879) (Figure 1).

### 2.2. Study Outcomes and Covariates

The definition of TOO within our study encompasses a series of meticulously selected criteria reflective of optimal post-surgical outcomes, which is based on the previously published literature describing textbook outcomes in surgical oncology [7,8,9,10,11]. These include achieving a negative (R0) resection margin, indicating no residual microscopic tumor; the examination of at least 12 lymph nodes to ensure thorough nodal staging [12]; the absence of a prolonged hospital stay beyond the 75th percentile of duration for this patient cohort (corresponding to >8 days); no instances of 30-day postoperative mortality; and the lack of any readmissions within 30 days post-surgery.

These criteria collectively serve as benchmarks for high-quality surgical care in the treatment of pancreatic adenocarcinoma. We further examined a range of covariates, including demographics, clinical factors (comorbidity, tumor, and surgery), and facility-specific data (volume, type). Hospital volume was defined as the annual number of oncologic pancreatic cases within each facility in the corresponding year of diagnosis. Facilities were then categorized into quartiles based on dataset-derived cutoffs and grouped: low-volume centers (≤23 cases/year), moderate-volume centers (between 24 and 50 cases/year), high-volume centers (between 51 and 103 cases/year), and highest-volume centers (104+ cases/year).

### 2.3. Statistical Analysis

**Figure A1:**
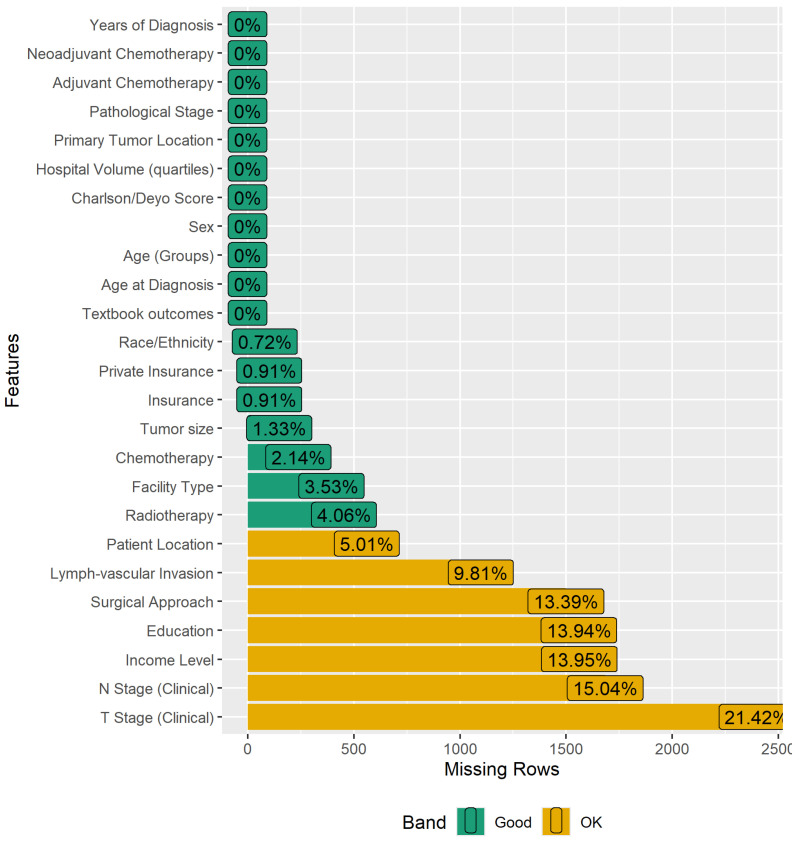
Percentages of missing data from characteristics of our patients and the variables used for modeling.

Patient characteristics were summarized using descriptive statistics. Continuous variables were reported as means (standard deviation, SD) or medians (interquartile range, IQR) following normality assessment. Categorical variables were described using frequencies and percentages. Differences between the TOO+ and TOO− patient groups were evaluated using the Wilcoxon rank sum test for continuous variables and Pearson’s χ2 or Fisher’s exact tests for categorical variables. Univariable and multivariable logistic regression analyses were conducted to identify factors associated with TOO achievement. In cases where factors were significant in the univariable models and became non-significant in the multivariable models, a sensitivity analysis was conducted to explore potential factors that caused this discrepancy. Continuous variables were standardized using Z scores. Missing data (Appendix A, Figure A1) were handled using random forest-based imputation, through iterative imputation using non-parametric models that capture complex, nonlinear relationships between variables without relying on distributional assumptions using 100 trees and 6 iterations. Correlation among variables was checked to inform the regression analysis. Overall survival (OS) was assessed with the Kaplan–Meier method, and differences in survival between the main groups (TOO+ vs. TOO−) were examined using the log-rank test. Factors associated with overall survival were assessed using univariable and multivariable Cox regression analyses were conducted. All statistical analyses were performed in R (version 4.5.1 [2025-06-13 ucrt)]), using ‘gtsummary’, ‘missRanger’, and ‘survminer’ packages [13,14,15], along with their dependencies. Two-sided *p*-values and 95% confidence intervals (CI) were reported, with significance considered at α = 0.05.

## 3. Results

### 3.1. Patient Demographics and Clinical Characteristics

A total of 11,194 patients who underwent distal pancreatectomy for pancreatic adenocarcinoma between 1 January 2010 and 31 December 2022 were included in the analysis (2022 data without known follow-up, n = 879, were excluded). The mean age at diagnosis was 67 years (SD 12), with 52% female. The majority of patients had tumors located in the tail (61%) versus the body (39%) of the pancreas. Approximately 38.9% (n = 4350) of patients achieved TOO following surgery. Among the patients who achieved TOO (TOO+, N = 4350), there were no significant differences in pathological stage with TOO− patients. TOO+ were more likely to present from areas of higher education, income, and highly populated places (*p* < 0.001). They are presented with lower Charlson/Deyo scores, with private insurance, and received treatment at academic and highest-volume centers (*p* < 0.001). Additionally, a higher proportion of TOO+ patients received adjuvant chemotherapy (70% vs. 50%, *p* < 0.001) but less radiotherapy (18% vs. 22%) than TOO− patients. Table 1 summarizes the basic demographic results and compares the TOO+ and TOO− subgroups.

In our cohort, among patients not achieving TOO (TOO−, N = 6844), the most common reasons for failure were inadequate lymph node sampling (40%) and prolonged length of stay (35%), followed by positive margins (27%), readmissions (15%), and 30-day mortality (1.3%) (Table 2). Note: Percentages are calculated within the TOO− cohort and, since patients may have more than one reason for failure, percentages do not sum to 100%. 

### 3.2. Overall Survival Analysis

**Figure 2:**
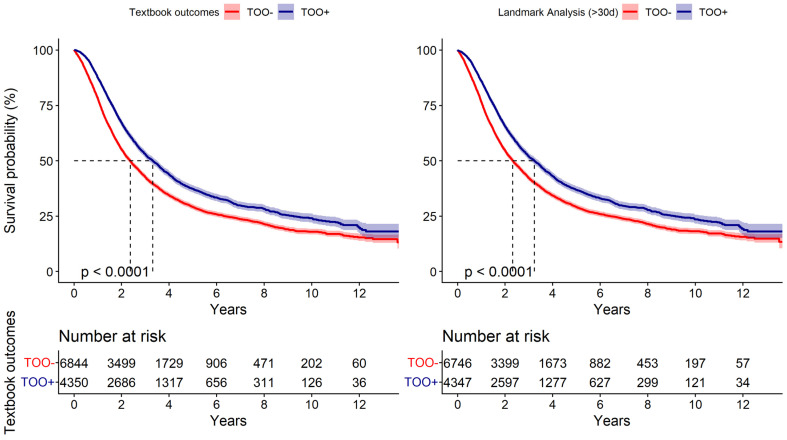
Kaplan–Meier survival curves for patients undergoing a distal pancreatectomy for pancreatic adenocarcinoma, stratified by achievement of textbook oncologic outcomes (TOO). Description: This figure presents the Kaplan–Meier survival curves for all patients (**left**) comparing the overall survival (OS) of patients undergoing a distal pancreatectomy for pancreatic adenocarcinoma, stratified into two groups based on the achievement of textbook oncologic outcomes (TOO). Landmark survival curves solely including patients alive at 30 days (**right**), with time re-indexed from the landmark, supported that the survival advantage associated with TOO remained consistent with those surviving the perioperative period.

In total, 7386 patients of the cohort passed away over the course of 14 years. The 1-year, 5-year, and 10-year survival rates were 82% (95% CI: 81%, 82%), 32% (95% CI: 31%, 33%), and 20% (95% CI: 19%, 21%), respectively, with a median overall survival (OS) of 2.7 years (95% CI: 2.6, 2.8). TOO+ patients demonstrated improved survival rates (Figure 2, left panel) at 1-year (88%), 5-years (37%), and 10-years (24%), with a median OS of 3.9 years (95% CI: 3.7, 4.1) compared to TOO− (78%, 29%, and 18%, respectively, with a median OS of 2.4 years and 95% CI: 2.3, 2.4). (Appendix A Table A1) A 30-day landmark analysis demonstrated that the survival advantage associated with achieving TOO persisted beyond the early postoperative period (Figure 2, right panel), suggesting that this association is not solely attributable to early mortality differences.

### 3.3. Factors Associated with TOO Achievement

Univariable analysis identified female sex, private insurance, lower Charlson/Deyo score, minimally invasive surgery (MIS), and treatment at the highest-volume hospitals as significantly associated with achieving TOO (all *p* < 0.05). Multivariable logistic regression confirmed these as independent predictors. Female sex was associated with higher odds of achieving TOO (OR 1.15, 95% CI: 1.05–1.26, *p* = 0.003), as was private insurance (OR 1.17, 95% CI: 1.05–1.13, *p* = 0.005). A Charlson/Deyo score of 1 compared to 0 was associated with lower odds of TOO (OR 0.89, 95% CI: 0.80–0.99, *p* = 0.029). MIS was independently associated with improved TOO achievement (OR 1.26, 95% CI: 1.14–1.40, *p* < 0.001). Higher T stage was associated with progressively lower odds of achieving TOO, with T3 (OR 0.74, 95% CI: 0.63–0.86, *p* < 0.001) and T4 disease (OR 0.62, 95% CI: 0.46–0.82, *p* = 0.001), demonstrating significantly reduced likelihood compared to T1.

Treatment at the highest-volume hospitals was associated with the highest rate of TOO achievement, with patients treated in these settings having significantly higher odds of achieving TOO (OR 2.12, 95% CI: 1.76–2.55, *p* < 0.001). Table 3 summarizes the logistic regression results. Hospital types, including academic, integrated, and community programs, showed no significant association with TOO in the multivariable model. However, the sensitivity analysis confirmed the significance of hospital type after excluding hospital volume, with integrated network, comprehensive community, and community cancer programs remaining significantly associated with lower odds of TOO achievement compared to academic centers.

### 3.4. Predictors of Overall Survival

Univariable Cox analysis showed that achievement of TOO, younger age, female sex, having private insurance, lower Charlson/Deyo score, MIS, adjuvant chemotherapy and treatment at higher-volume hospitals were associated with improved overall survival (all *p* < 0.05).

In the multivariable model, adjusting for clinical, tumor-related and treatment variables, achievement of TOO remained significantly associated with survival (HR 0.79, 95% CI: 0.74–0.83, *p* < 0.001). Factors associated with worse survival included age (HR 1.32, 95% CI: 1.27–1.36, *p* < 0.001), comorbidity burden (Charlson/Deyo score), nodal involvement, larger tumor size, lymphovascular invasion. More advanced disease stage was also strongly associated with mortality, with stage II (HR 1.63, 95% CI: 1.51–1.75, *p* < 0.001) and stage III (HR 2.25, 95% CI: 1.99–2.54, *p* < 0.001) showing progressively higher risk. In contrast, use of adjuvant chemotherapy (HR 0.78, 95% CI: 0.74–0.82, *p* < 0.001) and a minimally invasive surgical approach (HR 0.88, 95% CI: 0.83–0.93, *p* < 0.001) were associated with improved survival. Table 4 summarizes the Cox regression results.

## 4. Discussion

In our study assessing textbook oncologic outcomes (TOO) in distal pancreatectomies for pancreatic adenocarcinoma, significant findings emerged regarding the factors influencing TOO achievement and their association with overall survival (OS). This discussion aims to contextualize these findings within the broader landscape of pancreatic cancer treatment, highlighting their implications for surgical practice, patient counseling, and future research.

### 4.1. Implications of TOO Achievement

Achieving TOO represents a multifaceted measure of success in cancer surgery, encompassing complete tumor resection, adequate lymph node examination, absence of prolonged hospital stays, no 30-day mortality, and no readmissions. A study found that a significant portion of patients achieve TOO in distal pancreatectomy (38.9%) compared to pancreaticoduodenectomy, which was previously reported as 16.8% [16] in one study and 21.5% [17] in another. However, it was less than the 63–77% described by Petruch et al. in ductal adenocarcinoma, which includes both pancreaticoduodenectomy (84%) and distal pancreatectomy (16%), albeit with less-strict TOO parameters (prolonged hospital stay affected 35%, readmission affected 15%, and early mortality affected 1.3% of our population [Table 1]) [18]. Nevertheless, TOO had a similar association with improving survival, which demonstrates the critical importance of these composite outcomes in guiding surgical and postoperative care. The observed survival advantage among patients achieving TOO over time, as well as the one-year median survival benefit, highlights the tangible benefits of aiming for these comprehensive outcomes in surgical planning and execution. This survival benefit showcases the potential of TOO as a benchmark for quality in pancreatic cancer surgery, offering a more holistic evaluation of surgical success beyond traditional metrics.

### 4.2. Factors Associated with TOO Achievement

Several patient and institutional factors were identified as predictors of TOO achievement, including female sex, private insurance, lower Charlson/Deyo score, and treatment at high-volume hospitals. The association between these factors and TOO success points to underlying disparities in access to quality care and outcomes in pancreatic cancer treatment. Notably, the significant correlation between treatment at high-volume centers and increased TOO achievement highlights the importance of surgical expertise and institutional experience. This is consistent with previous studies indicating that high-volume centers tend to have better outcomes for complex surgical procedures due to specialized teams and more standardized postoperative care protocols. The discrepancy in hospital type significance before and after reporting the multivariable models highlights the confounding role of other factors, such as hospital volume. In the sensitivity analysis model integrated network, comprehensive community, and community cancer programs were significantly associated with lower odds of achieving TOO compared to academic centers. However, associations with hospital type lost significance in the full model, where hospital volume emerged as the strongest factor associated with TOO, which likely reflects confounding. This suggests that hospital type alone may not independently influence outcomes; instead, higher procedural volume and associated resources likely account for the observed differences in TOO achievement. Patients treated at high- and highest-volume centers (>50 and 103 cases/year, respectively) had significantly higher odds of achieving TOO compared to lower-volume centers, with the highest-volume centers showing over double the likelihood (OR 2.26, 95% CI: 1.93–2.64). This supports the claim that for large-scale surgeries, and in this specific case, distal pancreatectomy, increasing hospital case volume correlates with improved surgical outcomes.

### 4.3. Factors Associated with Overall Survival

In the adjusted analysis, achievement of TOO remained independently associated with improved survival, corresponding to roughly a 20% lower risk of mortality, persisted after accounting for differences in patient characteristics, tumor burden and treatment-related factors, suggesting that TOO may capture multiple aspects of surgical and perioperative care rather than simply reflecting baseline differences between patients. As expected, increasing age and more advanced disease were associated with worse outcomes, with stage III disease showing a substantially higher risk of mortality. Tumor-related features, such as larger tumor size and lymphovascular invasion, followed a similar pattern. In contrast, treatment-related factors, particularly with MIS and adjuvant chemotherapy, are associated with improved survival. While neoadjuvant was not statistically significant, it trended towards decreasing mortality risk. Taken together, these findings suggest that long-term outcomes are influenced not only by underlying disease severity but also by the aspects of surgical, oncologic, and perioperative care.

### 4.4. Role of Minimally Invasive Surgery

The study also revealed that minimally invasive surgery (MIS) was associated with higher TOO achievement rates. This finding contributes to the growing body of evidence supporting the benefits of MIS approaches [19], including reduced postoperative pain, shorter hospital stays, and quicker recovery, which can directly influence TOO metrics such as absence of prolonged hospital stays and no readmissions. The adoption of MIS techniques, where feasible, could therefore represent a strategic approach to improving TOO achievement rates in distal pancreatectomy for pancreatic adenocarcinoma. However, in cases requiring conversion to open surgery, there was no significant difference in TOO achievement compared to planned open procedures (OR 0.87, 95% CI: 0.75–1.01, *p* = 0.066). This suggests that while MIS offers advantages, conversion may neutralize some benefits, highlighting the importance of surgical expertise during the transition to MIS techniques. Thus, the decision to perform MIS will likely benefit achieving optimal outcomes that translate into better survival but also should be carefully considered in the context of tumor characteristics, patient health status, and surgeon expertise. Furthermore, this association extended to survival outcomes, as MIS remained independently associated with improved overall survival (HR 0.88, *p* < 0.001), suggesting that its benefits may go beyond perioperative recovery.

However, while conversion to open surgery was not statistically associated with survival (HR: 0.11, *p* = 0.10), it was associated with a significant reduction in TOO achievement (OR: HR 0.88, *p* = 0.036) compared to planned MIS. This is not only statistically significant but also clinically significant, as conversion often reflects intraoperative challenges such as adverse events, dense adhesions, or unfavorable anatomy, which are associated with longer operative times, increased complication risk, prolonged hospital stays, and higher readmission rates, all of which are factors that directly compromise TOO achievement. Thus, conversion likely represents a marker of operative complexity. However, the results considering MIS should be interpreted cautiously, since there is likely a potential selection bias related to patient condition and surgeon expertise.

Emerging mechanistic evidence may further contextualize the observed association between surgical approach and outcomes. Mechanical stress applied to pancreatic tissue during surgery has been shown to activate mechanosensitive ion channels, including Piezo1 and downstream TRPV4 pathways, leading to intracellular calcium influx and cellular injury [20]. Experimental models suggest that such calcium overload can trigger mitochondrial dysfunction, premature enzyme activation, and acinar cell necrosis, processes that may contribute to postoperative complications. Additionally, transient receptor potential (TRP) channels have been implicated in pancreatic ductal adenocarcinoma biology, including tumor progression and stromal interactions [21]. MIS may reduce direct tissue manipulation and mechanical stress, potentially attenuating these pathways and contributing to improved perioperative and oncologic outcomes. While speculative, this provides a biologically plausible explanation for the observed association between MIS and improved TOO achievement.

### 4.5. Challenges and Future Directions

Despite these insights, challenges remain in universally achieving TOO across diverse patient populations and healthcare settings. The disparities in TOO achievement related to demographic and insurance factors highlight systemic barriers that may limit access to high-quality surgical care. Addressing these disparities requires a multifaceted approach, including policy interventions, patient education, and efforts to broaden the availability of specialized care.

Further research is essential to explore strategies for improving TOO achievement rates, particularly among underrepresented and disadvantaged patient groups, as we observed that many patients who achieved TOO+ come from higher education, income, and highly populated places. This may highlight the association of social determinants of health on TOO, as was described in non-small-cell lung cancer before [4] and on esophageal cancer [22]. Additionally, studies examining the impact of emerging technologies and surgical techniques on TOO metrics can provide valuable information to refine surgical practices continuously.

### 4.6. Limitations

Several limitations should be considered when interpreting our findings. First, this study is retrospective in nature and subject to inherent selection bias and residual confounding, which limit causal inference. Although our multivariable models are adjusted for key clinical, tumor-specific, and treatment-related variables, unmeasured confounders, such as surgeon-level experience, intraoperative decision-making, detailed perioperative management, and institutional treatment protocols, are not captured in the database. Second, our observed association between minimally invasive surgery and improved outcomes may be influenced by selection bias, as patients selected for MIS are often healthier and have more favorable tumor characteristics. Third, the use of the NCDB limits the granularity of available data, including the lack of detailed information on postoperative complications (e.g., clinically relevant postoperative pancreatic fistula), recurrence patterns (local vs. distant), and quality-of-life or functional outcomes, all of which may influence textbook oncologic outcomes. Fourth, while we employed a random forest-based imputation method to address missing data, imputation may still introduce bias depending on the underlying mechanism of missingness and the variables used in the imputation process. Finally, as with all national registry-based studies, the data are subject to potential coding errors and unmeasured confounding that may not be reproducible.

## 5. Conclusions

The study’s findings illuminate the significance of TOO in distal pancreatectomy for pancreatic adenocarcinoma, demonstrating a clear association between TOO achievement and improved patient outcomes. By highlighting the factors influencing TOO success and the survival advantage conferred by achieving these outcomes, this research contributes to a deeper understanding of quality metrics in pancreatic cancer treatment. Moving forward, efforts to enhance TOO achievement rates must consider both clinical and systemic factors, aiming to elevate the standard of care and improve survival for patients with pancreatic adenocarcinoma.

## Figures and Tables

**Table 1:** Baseline characteristics of stage I–III pancreatic adenocarcinoma patients undergoing a distal pancreatectomy who achieved TOO vs. without TOO.

Characteristic	Overall N = 11,194 ^1^	TOO+ N = 4350 ^1^	TOO− N = 6844 ^1^	*p*-Value ^2^
Age at Diagnosis	67 (12)	67 (12)	67 (13)	**0.002**
Age (Groups)				**<0.001**
<50	887 (7.9%)	309 (7.1%)	578 (8.4%)	
50–59	1714 (15%)	757 (17%)	957 (14%)	
60–69	3468 (31%)	1372 (32%)	2096 (31%)	
70–79	3631 (32%)	1387 (32%)	2244 (33%)	
80+	1494 (13%)	525 (12%)	969 (14%)	
Sex				**<0.001**
Female	5804 (52%)	2378 (55%)	3426 (50%)	
Male	5390 (48%)	1972 (45%)	3418 (50%)	
Race/Ethnicity				0.13
White	8890 (79%)	3505 (81%)	5385 (79%)	
Hispanic	604 (5.4%)	228 (5.2%)	376 (5.5%)	
Black	1222 (11%)	438 (10%)	784 (11%)	
Asian	368 (3.3%)	141 (3.2%)	227 (3.3%)	
Other	110 (1.0%)	38 (0.9%)	72 (1.1%)	
Education				**0.001**
High	2408 (22%)	1004 (23%)	1404 (21%)	
Low	8786 (78%)	3346 (77%)	5440 (79%)	
Income Level				**<0.001**
>150% FPL	3946 (35%)	1617 (37%)	2329 (34%)	
100–150% FPL	5799 (52%)	2253 (52%)	3546 (52%)	
<100% FPL	1449 (13%)	480 (11%)	969 (14%)	
Patient Location				**<0.001**
Metro	9674 (86%)	3826 (88%)	5848 (85%)	
Urban	1334 (12%)	458 (11%)	876 (13%)	
Rural	186 (1.7%)	66 (1.5%)	120 (1.8%)	
Charlson/Deyo Score				**0.010**
0	6785 (61%)	2720 (63%)	4065 (59%)	
1	2917 (26%)	1086 (25%)	1831 (27%)	
2	868 (7.8%)	320 (7.4%)	548 (8.0%)	
3	624 (5.6%)	224 (5.1%)	400 (5.8%)	
Insurance				**<0.001**
Not Insured	162 (1.4%)	56 (1.3%)	106 (1.5%)	
Medicare/Medicaid/Other Government	7498 (67%)	2811 (65%)	4687 (68%)	
Private Insurance	3534 (32%)	1483 (34%)	2051 (30%)	
Facility Type				**<0.001**
Academic/Research Program	6098 (54%)	2585 (59%)	3513 (51%)	
Integrated Network Cancer Program	2018 (18%)	730 (17%)	1288 (19%)	
Comprehensive Community Cancer Program	2804 (25%)	941 (22%)	1863 (27%)	
Community Cancer Program	274 (2.4%)	94 (2.2%)	180 (2.6%)	
Hospital Volume (quartiles)				**<0.001**
Highest	4183 (37%)	2027 (47%)	2156 (32%)	
High	3350 (30%)	1233 (28%)	2117 (31%)	
Medium	2327 (21%)	692 (16%)	1635 (24%)	
Low	1334 (12%)	398 (9.1%)	936 (14%)	
Primary Tumor Location				**0.010**
Body	4317 (39%)	1742 (40%)	2575 (38%)	
Tail	6877 (61%)	2608 (60%)	4269 (62%)	
Overall Stage				0.7
Stage I	2965 (26%)	1169 (27%)	1796 (26%)	
Stage II	7461 (67%)	2877 (66%)	4584 (67%)	
Stage III	768 (6.9%)	304 (7.0%)	464 (6.8%)	
Surgical Approach				**<0.001**
Open	6792 (61%)	2485 (57%)	4307 (63%)	
MIS	3504 (31%)	1556 (36%)	1948 (28%)	
Conversion	898 (8.0%)	309 (7.1%)	589 (8.6%)	
Radiotherapy	2291 (20%)	763 (18%)	1528 (22%)	**<0.001**
Chemotherapy	7879 (70%)	3332 (77%)	4547 (66%)	**<0.001**

^1^ Mean (SD); n (%); ^2^ Wilcoxon rank sum test; Pearson’s Chi-squared test. Bold indicates significance *p* < 0.05.

**Table 2:** Reasons for failure to achieve textbook oncologic outcomes in stage I–III pancreatic adenocarcinoma patients undergoing a distal pancreatectomy.

Characteristic	TOO−, N = 6844 ^1^
30-Day Mortality	87 (1.3%)
30-Day Readmission	1013 (15%)
Residual Tumor	1822 (27%)
Long Length of Stay	2411 (35%)
12+ Regional Lymph Node Examined	4085 (60%)

^1^ n (%).

**Table 3:** Factors associated with TOO achievement in stage I–III distal pancreatectomy (unadjusted, sensitivity, and full multivariable logistic models).

	Univariable Module			Sensitivity Multivariable Module			Multivariable Module		
Characteristic	OR	95% CI	*p*-Value	OR	95% CI	*p*-Value	OR	95% CI	*p*-Value
Age (Groups)									
<50	—	—							
50–59	1.48	1.25, 1.75	**<0.001**						
60–69	1.22	1.05, 1.43	**0.010**						
70–79	1.16	0.99, 1.35	0.064						
80+	1.01	0.85, 1.21	0.9						
Age (Z Score)	0.97	0.94, 1.01	0.14	0.99	0.94, 1.05	0.7	0.99	0.94, 1.05	0.8
Female Sex	1.20	1.11, 1.30	**<0.001**	1.15	1.05, 1.26	**0.003**	1.15	1.05, 1.26	**0.003**
Private Insurance	1.21	1.11, 1.31	**<0.001**	1.19	1.06, 1.33	**0.003**	1.17	1.05, 1.31	**0.005**
Charlson/Deyo Score									
0	—	—		—	—		—	—	
1	0.89	0.81, 0.97	**0.008**	0.89	0.80, 0.99	**0.036**	0.89	0.80, 0.99	**0.029**
2	0.87	0.75, 1.01	0.068	0.92	0.77, 1.09	0.3	0.91	0.77, 1.09	0.3
3	0.84	0.70, 0.99	**0.041**	0.82	0.68, 1.00	0.055	0.81	0.67, 0.99	**0.042**
Surgical Approach									
Open	—	—		—	—		—	—	
MIS	1.38	1.27, 1.50	**<0.001**	1.30	1.18, 1.44	**<0.001**	1.26	1.14, 1.40	**<0.001**
Conversion	0.91	0.78, 1.05	0.2	0.86	0.72, 1.02	0.088	0.83	0.70, 0.99	**0.036**
Adjuvant Chemotherapy	1.57	1.45, 1.70	**<0.001**	1.60	1.45, 1.76	**<0.001**	1.61	1.46, 1.77	**<0.001**
Neoadjuvant Chemotherapy	1.16	1.02, 1.31	**0.023**	1.39	1.19, 1.61	**<0.001**	1.33	1.15, 1.55	**<0.001**
Facility Type									
Academic/Research Program	—	—		—	—		—	—	
Integrated Network Cancer Program	0.74	0.67, 0.82	**<0.001**	0.70	0.63, 0.79	**<0.001**	0.90	0.79, 1.02	0.11
Comprehensive Community Cancer Program	0.67	0.61, 0.73	**<0.001**	0.64	0.57, 0.71	**<0.001**	0.90	0.79, 1.03	0.13
Community Cancer Program	0.69	0.54, 0.89	**0.005**	0.65	0.48, 0.87	**0.004**	1.07	0.77, 1.48	0.7
Years of Surgery (Z Score)	1.25	1.21, 1.30	**<0.001**	1.27	1.21, 1.33	**<0.001**	1.24	1.18, 1.30	**<0.001**
T Stage (Clinical)									
T1	—	—		—	—		—	—	
T2	0.86	0.77, 0.97	**0.011**	0.90	0.79, 1.03	0.13	0.89	0.78, 1.02	0.087
T3	0.70	0.61, 0.79	**<0.001**	0.76	0.65, 0.88	**<0.001**	0.74	0.63, 0.86	**<0.001**
T4	0.65	0.51, 0.83	**<0.001**	0.64	0.48, 0.85	**0.002**	0.62	0.46, 0.82	**0.001**
N Stage (Clinical)									
N0	—	—		—	—		—	—	
N1	1.01	0.89, 1.15	0.9	1.18	1.02, 1.36	**0.024**	1.19	1.03, 1.37	**0.019**
N2	1.54	0.66, 3.60	0.3	1.52	0.63, 3.65	0.3	1.50	0.62, 3.64	0.4
Tumor size									
<2 cm	—	—		—	—		—	—	
2–3.9 cm	1.03	0.92, 1.17	0.6	1.08	0.92, 1.27	0.4	1.09	0.93, 1.28	0.3
4–7 cm	0.83	0.73, 0.94	**0.003**	0.91	0.76, 1.09	0.3	0.94	0.79, 1.13	0.5
>7 cm	0.58	0.48, 0.69	**<0.001**	0.67	0.53, 0.84	**<0.001**	0.68	0.54, 0.86	**0.001**
Pathological Stage									
Stage I	—	—		—	—		—	—	
Stage II	0.96	0.88, 1.05	0.4	1.17	1.04, 1.31	**0.011**	1.17	1.03, 1.31	**0.012**
Stage III	1.01	0.86, 1.18	>0.9	1.05	0.86, 1.30	0.6	1.04	0.84, 1.28	0.7
Lymph-vascular Invasion									
Not Present	—	—		—	—		—	—	
Present	1.05	0.97, 1.13	0.2	1.00	0.91, 1.10	>0.9	0.97	0.89, 1.07	0.6
Hospital Volume									
Low	—	—					—	—	
Medium	1.00	0.86, 1.15	>0.9				1.06	0.88, 1.27	0.6
High	1.37	1.20, 1.57	**<0.001**				1.28	1.08, 1.54	**0.006**
Highest	2.21	1.94, 2.53	**<0.001**				2.12	1.76, 2.55	**<0.001**

Abbreviations: CI = Confidence Interval, OR = Odds Ratio. Bold indicates significance *p* < 0.05.

**Table 4:** Factors associated with overall survival in stage I–III distal pancreatectomies (unadjusted, sensitivity, and full multivariable cox models).

	Univariable Module			Sensitivity Multivariable Module			Multivariable Module		
Characteristic	HR	95% CI	*p*-Value	HR	95% CI	*p*-Value	HR	95% CI	*p*-Value
TOO	0.75	0.72, 0.79	**<0.001**	0.79	0.74, 0.83	**<0.001**	0.79	0.74, 0.83	**<0.001**
Age (Z Score)	1.35	1.32, 1.38	**<0.001**	1.31	1.26, 1.35	**<0.001**	1.32	1.27, 1.36	**<0.001**
Female Sex	0.79	0.75, 0.82	**<0.001**	0.91	0.87, 0.96	**<0.001**	0.92	0.87, 0.97	**0.003**
Private Insurance	0.72	0.68, 0.76	**<0.001**	0.95	0.89, 1.02	0.2	0.96	0.90, 1.02	0.2
Charlson/Deyo Score									
0	—	—		—	—		—	—	
1	1.20	1.14, 1.27	**<0.001**	1.13	1.06, 1.20	**<0.001**	1.13	1.06, 1.20	**<0.001**
2	1.42	1.31, 1.55	**<0.001**	1.31	1.19, 1.45	**<0.001**	1.30	1.18, 1.43	**<0.001**
3	1.54	1.40, 1.69	**<0.001**	1.38	1.24, 1.54	**<0.001**	1.41	1.26, 1.56	**<0.001**
Surgical Approach									
Open	—	—		—	—		—	—	
MIS	0.81	0.77, 0.85	**<0.001**	0.88	0.83, 0.93	**<0.001**	0.88	0.83, 0.93	**<0.001**
Conversion	1.06	0.98, 1.16	0.15	1.08	0.98, 1.19	0.11	1.08	0.98, 1.19	0.10
Adjuvant Chemotherapy	0.93	0.89, 0.98	**0.003**	0.78	0.74, 0.83	**<0.001**	0.78	0.74, 0.82	**<0.001**
Neoadjuvant Chemotherapy	1.02	0.95, 1.10	0.5	0.91	0.84, 0.99	**0.029**	0.92	0.84, 1.00	0.051
Facility Type									
Academic/Research Program	—	—		—	—		—	—	
Integrated Network Cancer Program	1.01	0.95, 1.08	0.7	1.10	1.02, 1.19	**0.009**	1.10	1.02, 1.18	**0.013**
Comprehensive Community Cancer Program	1.21	1.14, 1.28	**<0.001**	1.09	1.02, 1.18	**0.017**	1.08	1.01, 1.16	**0.036**
Community Cancer Program	1.34	1.16, 1.55	**<0.001**	1.27	1.06, 1.51	**0.010**	1.24	1.04, 1.48	**0.018**
Years of Surgery (Z Score)	0.86	0.83, 0.88	**<0.001**	0.88	0.86, 0.91	**<0.001**	0.88	0.86, 0.91	**<0.001**
T Stage (Clinical)									
T1	—	—		—	—		—	—	
T2	1.31	1.21, 1.41	**<0.001**	1.18	1.09, 1.28	**<0.001**	1.04	0.96, 1.13	0.3
T3	1.60	1.47, 1.73	**<0.001**	1.23	1.13, 1.34	**<0.001**	1.08	0.98, 1.18	0.11
T4	1.57	1.37, 1.79	**<0.001**	1.20	1.03, 1.40	**0.019**	1.05	0.90, 1.23	0.6
N Stage (Clinical)									
N0	—	—		—	—		—	—	
N1	1.44	1.34, 1.55	**<0.001**	1.22	1.13, 1.32	**<0.001**	1.22	1.13, 1.32	**<0.001**
N2	1.08	0.60, 1.94	0.8	0.84	0.46, 1.52	0.6	0.83	0.46, 1.52	0.6
Tumor size									
<2 cm	—	—					—	—	
2–3.9 cm	1.72	1.58, 1.87	**<0.001**				1.40	1.25, 1.55	**<0.001**
4–7 cm	2.25	2.07, 2.45	**<0.001**				1.60	1.43, 1.79	**<0.001**
>7 cm	1.88	1.68, 2.09	**<0.001**				1.52	1.33, 1.75	**<0.001**
Pathological Stage									
Stage I	—	—		—	—		—	—	
Stage II	2.22	2.09, 2.36	**<0.001**	1.73	1.60, 1.86	**<0.001**	1.63	1.51, 1.75	**<0.001**
Stage III	3.01	2.72, 3.32	**<0.001**	2.41	2.13, 2.72	**<0.001**	2.25	1.99, 2.54	**<0.001**
Lymph-vascular Invasion									
Not Present	—	—		—	—		—	—	
Present	1.56	1.49, 1.64	**<0.001**	1.34	1.27, 1.41	**<0.001**	1.32	1.25, 1.39	**<0.001**
Hospital Volume									
Low	—	—		—	—		—	—	
Medium	0.90	0.83, 0.98	**0.013**	1.01	0.92, 1.12	0.8	1.00	0.91, 1.11	>0.9
High	0.82	0.76, 0.89	**<0.001**	0.98	0.89, 1.08	0.7	0.98	0.89, 1.08	0.6
Highest	0.73	0.68, 0.78	**<0.001**	0.85	0.77, 0.94	**0.002**	0.85	0.77, 0.94	**0.002**

Abbreviations: CI = Confidence Interval, HR = Hazard Ratio. Bold indicates significance *p* < 0.05.

## Data Availability

The raw data supporting the findings of this study are available from the National Cancer Database (NCDB) and may be accessed by investigators at Commission on Cancer–accredited institutions through the American College of Surgeons. Due to data use agreements and privacy restrictions, the data are not publicly available. All analyses were conducted using reproducible statistical code, which can be requested from the corresponding author, A.A.

## Appendix A

**Table A1:** Survival rates at 1, 5, and 10 years and median survival time of stage I–III pancreatic adenocarcinoma patients.

Characteristic	1 Year	5 Year	10 Year	Median Survival
Overall	82% (81%, 82%)	32% (31%, 33%)	20% (19%, 21%)	2.7 (2.6, 2.8)
Textbook Outcomes				
TOO−	78% (77%, 79%)	29% (28%, 30%)	18% (17%, 19%)	2.4 (2.3, 2.4)
TOO+	88% (87%, 89%)	37% (36%, 39%)	24% (22%, 26%)	3.3 (3.1, 3.5)
Surgical Approach				
Open	80% (79%, 81%)	30% (29%, 31%)	19% (18%, 20%)	2.5 (2.4, 2.6)
MIS	86% (85%, 87%)	38% (36%, 40%)	24% (22%, 27%)	3.2 (3.0, 3.4)
Conversion	79% (76%, 82%)	28% (25%, 32%)	15% (12%, 20%)	2.4 (2.2, 2.7)
Hospital Volume				
Low	76% (74%, 78%)	26% (24%, 29%)	16% (13%, 19%)	2.1 (1.9, 2.3)
Medium	78% (77%, 80%)	29% (27%, 32%)	19% (17%, 21%)	2.4 (2.2, 2.5)
High	81% (79%, 82%)	33% (31%, 34%)	20% (18%, 22%)	2.7 (2.6, 2.9)
Highest	86% (85%, 87%)	36% (34%, 37%)	23% (21%, 25%)	3.1 (3.0, 3.3)
